# A Possible Future in Medical, Psycho-Social and Moral Fields

**DOI:** 10.25122/jml-2020-1001

**Published:** 2020

**Authors:** Victor Lorin Purcarea

Motto: Overcoming fear is the beginning of wisdom - Bertrand Russell

Philosopher, mathematician, historian, etc. Bertrand Russel, who acknowledged that one of the three passions that governed his life was boundless pity for the suffering of mankind, affirmed, more than half a century ago: “*Maybe the universe itself has a purpose, but, even if it were, nothing suggests that it would be similar to ours*”.

He was right. And it was because now we are going through an unexpected, unwanted, unimagined and unforeseen period!

The risks that humanity face at present are increasing as the world becomes more interconnected, the COVID-19 *pandemic* not stopping at the borders, affecting people regardless of nationality, training, education, income or gender.

The reaction of the people lost in the sphere of daily finding the personal comfort and removed from the turmoil of searching for this ephemeral and eternal insufficient comfort was and still is aberrant, because the pandemic required a change. And the reaction to this change, so necessary for us, was and is disconcerting.

If China was the first country hit by COVID-19 and it reacted rapidly and efficiently, the next affected countries had different reactions, and the consequences were, unfortunately, dramatic.

In our country, the fight with COVID-19 virus has been accompanied by several “parallel fights”, among which the fight with the people's *fear*, which, in many places, has turned into *panic* – one of the most dangerous social phenomena, because reasoning does not help the panicked people anymore, the trust in God or the state authority disappears and they are capable of anything.

Along with the war against the new COVID-19, there are also other wars: against the *pseudo-news* and the* lack of responsibility*, a lot of irresponsible people becoming the enemies of the responsible people, whose health and even lives they endanger.

Pseudo-news, the so-called “fake news” resemble the new coronavirus: they spread extremely fast, faster than most of the news.

The lack of responsibility generates dangerous behavioral slippages, many people reacting to official communications with explanations from the area of conspiracy theory on a social background in which people simply do not trust institutions anymore, neither public nor media institutions. And this happens despite the fact that even though the decision-making authorities’ effort is beginning to become more and more visible by increased number and speed of epidemiological investigations, strengthening of Public Health Departments, including the participation of resident doctors, and huge efforts of quarantine and isolation and hospitals’ protection are made by urgently procuring the necessary materials to protect the employees and patients.

But what will the future hold? Maybe the following:

1. Unification of the medical system at the level of the European Union (specializations, procedures, protocols, hospital capacities), a process that will make possible a better *interoperability*, deficient during the *pandemic*, more *solidarity*, greater *unity*, as well as a better coordination at the community level.
2. The health system will be reformed from the ground, from resources to management, in the context of redefining the social assistance policy; other relationships will be born at workplace and the traditional hierarchies will be overturned – largely based on professional and human experiences formed during the *pandemic*.
3. The health system and the education system will be reassessed at the social level, which will be financed more then before, because the *pandemic* has revealed not only the *human* importance but also the *strategic* importance of the two systems: the vulnerability in logistics and human resource of the health system and the lack of moral education of the population, both being perceived as threats to national security.
4. The education will have other financial, logistical and human resources, which will allow the rise of a new way of teaching: on the one hand, the *on-line* learning strategies which were practiced during the *pandemic* by students of all ages and which many professors became acquainted with will develop, because home-education forced the latter to leave the “comfort zone” in which they used to be before; on the other hand, the educational process will be centered on the set of moral values that have been found not to define the *ethos* of Romanians: social solidarity, social responsibility and cooperation (a phenomenon perceived by the daily press as “a wave of stupidity, selfishness and insensitivity”).
5. Encouraging public investments in critical areas such as road and rail infrastructure will remove the vulnerabilities found at present and at the same time provide jobs to Romanian citizens who returned to the country during the *pandemic*, which will lead to unemployment reduction, increased purchasing power of the population, encouragement of consumption and development of production.
6. The state will become stronger due to an increased ability to react to events, anticipate crises and manage crisis situations: drastically simplifying the bureaucracy, modifying legislation on public procurement, strengthening the coordination capacity and achieving synergy at the level of all state institutions as well as closeness of the state to the citizen by redefining the principles of organization and functioning of its structures.
7. The overall rethinking of the state budget, not only in favor of health and education but also in favor of industries that have proved essential for the population's medical security: the pharmaceutical industry, the medical devices and personal protection devices industry, etc.
8. In the field of the apparent values, the following will lose ground in favor of things essential for human life: things considered hitherto urgent in favor of the perennial ones, defining for the human condition; positions in the prestigious hierarchy in favor of useful activities to the society on which our life and health depend; brand obsession in favor of the usefulness of things; snobbery in favor of modesty; consumer waste in favor of freely agreed austerity; social achievement in favor of honest work; careerism in favor of family relationships and friendship; egocentrism in favor of empathy; egoism in favor of altruism; material values in favor of spiritual ones.
9. Inherently, people “*enriched after the pandemic*” will appear, like the ones “after the war” and “after the Revolution”.
10. There will be a clear difference between people of character and the ones who only profit, and the former will form a new elite of the society in which people will have more confidence than they have in the current one. Returning to this reaction to the change at present, naturally, explainable but extremely harmful, of most of the people, one thing is obvious: *the need for health education*. Everyone needs education in the field of health in order to improve awareness and increase the level of interest in all segments of society, to preserve our lives by opening our minds, purifying our spirits, overcoming the fragmentation of understanding, interacting correctly, effectively engaging, constantly communicating and continuously learning how to make the appropriate change in our behavior.

The challenge in the permanent confrontation with life does not consist in defending or opposing social positions, functions or levels of social spending but in moral reconfiguration through consistent and real values.

But education is at the heart of adaptation!

